# Tick-borne pathogens in ticks collected from birds in Taiwan

**DOI:** 10.1186/s13071-017-2535-4

**Published:** 2017-11-25

**Authors:** Chi-Chien Kuo, Yi-Fu Lin, Cheng-Te Yao, Han-Chun Shih, Lo-Hsuan Chung, Hsien-Chun Liao, Yu-Cheng Hsu, Hsi-Chieh Wang

**Affiliations:** 1 Department of Life Science, National Taiwan Normal University, Taipei, Taiwan; 2 Department of Life Science, National Chung Hsing University, Taichung, Taiwan; 3 Endemic Species Research Institute, Council of Agriculture, Chi-chi, Nantou, Taiwan; 4 Center for Diagnostics and Vaccine Development, Centers for Disease Control, Ministry of Health and Welfare, Taipei, Taiwan; 5 Department of Natural Resources and Environmental Studies, National Dong Hwa University, Hualien, Taiwan

**Keywords:** Birds, Migratory birds, Ticks, Tick-borne pathogens, Taiwan

## Abstract

**Background:**

A variety of human diseases transmitted by arthropod vectors, including ticks, are emerging around the globe. Birds are known to be hosts of ticks and can disperse exotic ticks and tick-borne pathogens. In Taiwan, previous studies have focused predominantly on mammals, leaving the role of birds in the maintenance of ticks and dissemination of tick-borne pathogens undetermined.

**Methods:**

Ticks were collected opportunistically when birds were studied from 1995 to 2013. Furthermore, to improve knowledge on the prevalence and mean load of tick infestation on birds in Taiwan, ticks were thoroughly searched for when birds were mist-netted at seven sites between September 2014 and April 2016 in eastern Taiwan. Ticks were identified based on both morphological and molecular information and were screened for potential tick-borne pathogens, including the genera *Anaplasma*, *Babesia*, *Borrelia*, *Ehrlichia* and *Rickettsia*. Finally, a list of hard tick species collected from birds in Taiwan was compiled based on past work and the current study.

**Results:**

Nineteen ticks (all larvae) were recovered from four of the 3096 unique mist-netted bird individuals, yielding a mean load of 0.006 ticks/individual and an overall prevalence of 0.13%. A total of 139 ticks from birds, comprising 48 larvae, 35 nymphs, 55 adults and one individual of unknown life stage, were collected from 1995 to 2016, and 11 species of four genera were identified, including three newly recorded species (*Haemaphysalis wellingtoni*, *Ixodes columnae* and *Ixodes turdus*). A total of eight tick-borne pathogens were detected, with five species (*Borrelia turdi*, *Anaplasma* sp. clone BJ01, *Ehrlichia* sp. BL157-9, *Rickettsia helvetica* and *Rickettsia monacensis*) not previously isolated in Taiwan. Overall, 16 tick species of five genera have been recorded feeding on birds, including nine species first discovered in this study.

**Conclusion:**

Our study demonstrates the paucity of information on ticks of birds and emphasizes the need for more research on ticks of birds in Taiwan and Southeast Asia. Moreover, some newly recorded ticks and tick-borne pathogens were found only on migratory birds, demonstrating the necessity of further surveillance on these highly mobile species.

**Electronic supplementary material:**

The online version of this article (10.1186/s13071-017-2535-4) contains supplementary material, which is available to authorized users.

## Background

Ticks transmit the largest number of pathogens among all arthropod disease vectors and are second only to mosquitoes in their significance for human health. Moreover, several tick-borne diseases are expanding rapidly, such as anaplasmosis, babesiosis, Lyme disease, spotted fever and tick-borne encephalitis [1–4].

Ticks typically have four life stages: egg, larva, nymph and adult; a single blood meal from vertebrates is necessary for the larva and nymph to molt into the next life stage and for the adult female to lay eggs [5]. Small mammals are often one of the primary hosts for immature ticks [6], but a growing number of studies have revealed the significance of birds as hosts, as well as the role migratory birds play in the long-distance dissemination of ticks and tick-borne pathogens [7–13]. For example, immature western black-legged ticks *Ixodes pacificus*, the principal vector responsible for *Borrelia burgdorferi* (*sensu stricto*) in California, can be found on more avian than mammalian species [14]. Likewise, a large number of bird species are hosts of *Ixodes scapularis* [15]. Many tick species have been found on migratory birds [16–21], demonstrating their ability to spread ticks over long distances. More importantly, pathogens transmitted by ticks might be imported through the migration of birds. For example, the tick-borne spotted fever group (SFG) rickettsiae have been detected in exotic ticks recovered from migratory birds in Louisiana [21] and Russia [22]. Migratory birds have also been implicated in the spread of a variety of tick-borne diseases by carrying pathogen-infected ticks, including Lyme disease and Lyme borreliosis [7, 23, 24], tick-borne encephalitis [25], babesiosis [26], anaplasmosis [27] and Crimean-Congo hemorrhagic fever [28].

Although birds play a significant role in the subsistence of some ticks and tick-borne pathogens, their importance varies among species. For example, birds foraging primarily on the ground are more likely to acquire ticks than species foraging in trees and shrubs [29]. Species residing in dense oak woodland are more frequently infested with ticks than species living in chaparral, grass or a mixture of oak woodland and grass [30]. A review article found that in North America, non-migratory, ground-foraging birds are more likely to be carriers of ticks, and major tick carriers are almost all passerines [13]. Similarly, reservoir competence of *Borrelia burgdorferi* (*s.s*.), the etiological agent of Lyme disease, also differed greatly among avian species [31]. Identifying these principal avian host species involved in pathogen cycles can assist in a more effective control of tick-borne diseases.

In Taiwan, tick-borne pathogens that have been isolated from ticks include tick-borne SFG rickettsiae [32–35], *Anaplasma* and *Ehrlichia* [35], *Bartonella* [36–38], *Borrelia* spp. bacteria that might cause Lyme borreliosis [39, 40] and *Cytauxzoon* protozoans [35]. Ticks assayed in these studies, along with investigation or documentation of other tick-host associations in Taiwan [41–51], focused predominantly on mammals. The only study [52] that has focused on ticks of birds covered a large geographical area (Oriental, Palaearctic, Malagasy and Ethiopian regions) and provided little information on tick-bird associations in Taiwan. Knowledge of ticks on birds and the tick-borne pathogens that these ticks can harbor remains very limited, not only in Taiwan but also across Southeast Asia. In Malaysia, tick infestation has been examined for seven avian species, but no ticks have been found [53]. Similarly, 15 species of birds have been investigated in Thailand, but only four ticks have been collected on one avian species [54]. Information is also needed on the role of migratory birds in the importation of ticks and tick-borne pathogens that are likely of exotic origin. The aim of this study is to investigate tick infestation on birds in Taiwan, including both resident and migratory birds, and screen for pathogens harbored by these ticks. Ticks were collected from two sources: an opportunistic collection of ticks from mist-netted or wounded birds and a thorough examination of tick infestation on mist-netted birds; the latter was implemented to reveal the prevalence and load of tick infestation on birds. Finally, we updated the list of hard tick species (Ixodidae) collected from birds in Taiwan based on previous work and the current study.

## Methods

### Collection of ticks on birds

**Fig. 1:**
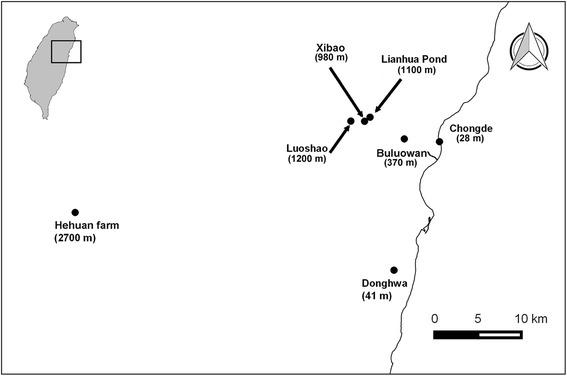
Sites for mist-netting birds in Taiwan from September 2014 to April 2016. Dominant habitats for each site: Chongde: fallow fields; Donghwa: shrubland; Buluowan: forest; Xibao: fallow fields; Lianhua Pond: shrubland; Luoshao: fallow fields; Hehuan farm: forest

Ticks of birds were collected opportunistically during two avian studies in which investigation of ectoparasites was not the main purpose; that is, ticks were collected when they were incidentally noticed by the researchers. The first study took place between 1995 and 2008, with birds being caught primarily by mist-netting around Taiwan in preparation for voucher specimens to be archived in the Endemic Species Research Institute of Taiwan. The collected ticks were preserved in 75% ethanol and were stored at room temperature. Among this collection of ticks, 12 have been morphologically identified and reported [51]. The other study took place during 2009–2013, with birds being mist-netted at seven sites in the Taroko National Park and its surrounding areas in eastern Taiwan (Fig. 1) for a long-term avian biodiversity monitoring project. These seven sites were (site name and elevation in meters above sea level) as follows: Chongde (28 m); Donghwa (41 m); Buluowan (370 m); Xibao (980 m); Lianhua Pond (1100 m); Luoshao (1200 m); and Hehuan Farm (2700 m) (Fig. 1). Because avian faunas vary with elevation in Taiwan [55], these sites, with an altitudinal difference of nearly 2700 m, covered different avian species. These study sites also included different habitat types, such as forests, farmlands, old fields and grassland. At each site, 6 to 10 mist nets were erected in the mornings and afternoons for two consecutive days, except for Donghwa, which was surveyed for two consecutive mornings only. Once captured, birds were banded, and a selection of morphological characteristics was measured. Ectoparasites, including ticks and lice, were collected when they were incidentally noticed. Ectoparasites were preserved in 100% ethanol and stored at -20 °C.

Because infested ticks might have been overlooked in the above-mentioned opportunistic collections, to improve knowledge of the prevalence (i.e. the number of infested birds divided by the total number of the bird individuals studied) and mean load (i.e. the total number of ticks divided by the total number of the bird individuals studied) of tick infestation on birds in Taiwan, from September 2014 to April 2016, birds mist-netted at the same seven study sites in eastern Taiwan (Fig. 1) were thoroughly (instead of opportunistically) examined for infestations of ticks. Ticks were also preserved in 100% ethanol and stored at -20 °C. The number of mist nets erected and days of survey at each study site were the same as for the 2009–2013 period. However, the frequency of surveys varied among the study sites due to the difficulty of accessing some sites or less sampling was implemented in sites with few birds trapped. Because migratory birds stopped over in Taiwan from September to April of the following year, the sampling period (September 2014 to April 2016) included two migratory seasons and a 20-month-long examination of resident birds. Wounded birds or birds accidently trapped by farmers during this survey period were also carefully examined for tick infestations. In this study, scientific names of birds and the division of migratory and resident birds follow Clements et al. [56].

### Identification of tick species

Ticks were examined under a dissecting microscope (Leica MZ12) and morphologically identified to species using published keys [57, 58]. When unrecognized, ticks were molecularly identified by comparing 12S rDNA and 16S rDNA sequences (primers provided in Table 1) with known species - following Black & Piesman [59] and Beati & Keirans [60]. The PCR products were purified using the QIAquick Gel Extraction Kit (Qiagen, Valencia, USA), and DNA sequencing was carried out using the ABI 3730XL DNA sequencer (Applied Biosystems, Foster City, USA) according to the manufacturer’s protocol. The PCR products were sequenced twice in each direction and were searched for resemblance to known tick species by using the Basic Local Alignment Search Tool (BLAST) [61]. Representative sequences are submitted in the GenBank database under accession numbers MG283136 (*Haemaphysalis wellingtoni*), MG283137 (*Ixodes columnae*), and MG283138 (*Ixodes turdus*).

**Table 1:** Primers for tick species and tick-borne pathogen detections in hard ticks (Ixodidae) of birds in Taiwan

	Gene target	Primers	Sequences (5′-3′)	Product size (bp)	Method	Reference
Tick species	12S rRNA gene	T1B	AAACTAGGATTAGATACCCT	379	PCR	[60]
		T2A	AATGAGAGCGACGGGCGATGT			
	16S rRNA gene	16S + 1	CTGCTCAATGATTTTTTAAATTGCTGTGG	452	PCR	[59]
		16S-1	CCGGTCTGAACTCAGATCAAGTA			
		BmR1	TGTTATTGCCTTACACTTCCTTGC			
		BmF2	ACGGCTACCACATCTAAGGAAGGC			
		BmR2	TCTCTCAAGGTGCTGAAGGA			
*Anaplasma* spp. and *Ehrlichia* spp.	16S rRNA gene	EHR 16SD	GGTACC(C/T)ACAGAAGAAGTCC	306	Real time PCR	[119]
		EHR 16SR	TAGCACTCATCGTTTACAGC			
*Rickettsia* spp.	OmpB	rompB OF	GTAACCGGAAGTAATCGTTTCGTAA	426 or 250	Nested PCR	[120]
		rompB OR	GCTTTATAACCAGCTAAACCACC			
		rompB SFG IF	GTTTAATACGTGCTGCTAACCAA			
		SFG/TG IR	GGTTTGGCCCATATACCATAAG			
		rompB TG IF	AAGATCCTTCTGATGTTGCAACA			
	gltA	RpCS.877p	GGGGGCCTGCTCACGGCGG	338	Nested PCR	
		RpCS.1258n	AATGCAAAAAGTACAGTGAACA			
		RpCS.896	GGCTAATGAAGCAGTGATAA			
		RpCS.1233n	GCGACGGTATACCCATAGC			
*Borrelia* spp.	rrf(5S)-rrl(23S)	5S–F	CGACCTTCTTCGCCTTAAAGC	226–266	Nested PCR	[121]
		23S–R	TAAGCTGACTAATACTAATTACCC			
		5S–rrf	CTGCGAGTTCGCGGGAGA			
		23S–rrl	TCCTAGGCATTCACCATA			
*Babesia* spp.	18S rRNA gene	BmF1	GCGATGTATCATTCAAGTTTCTG	700	Nested PCR	[122]
		BmR1	TGTTATTGCCTTACACTTCCTTGC			
		BmF2	ACGGCTACCACATCTAAGGAAGGC			
		BmR2	TCTCTCAAGGTGCTGAAGGA			

### Pathogen identification in ticks

Because there are very few collections of ticks of birds in Taiwan, and due to the necessity that ticks be destroyed to be assayed for pathogen infection, only a portion of tick samples was obtained for pathogen detection, with another portion of tick specimens being saved as voucher specimens. When a bird was infested with more than one tick of the same species (based on morphological identification), about half of the specimens were selected to be assayed for pathogen infection. We also investigated pathogens in those unrecognized ticks that required molecular species identification.

The screened tick-borne pathogens occur in Taiwan or in nearby countries along the bird migration routes. A total of five groups of pathogens (and the potentially resultant human diseases) were assayed: *Anaplasma* (causative agent of anaplasmosis), *Ehrlichia* (causative agent of ehrlichiosis), *Rickettsia* (SFG rickettsiae), *Borrelia* (causative agent of Lyme disease) and *Babesia* (causative agent of babesiosis). Primers and methods for amplifying fragments of these pathogens were provided in Table 1. The PCR protocol also followed the manufacturer’s instructions, and positive samples were sequenced to identify potential microbial species with a resemblance to known species based on BLAST. Representative sequences are submitted in the GenBank database under accession numbers MG434346 (*Borrelia turdi*), MG346222 (*Anaplasma* sp. clone BJ01) and MG346223 (*Ehrlichia* sp. BL157-9).

### Published tick species on birds in Taiwan

We compiled a list of hard ticks on birds in Taiwan based on published papers, books, theses and the current study. Papers were searched in PubMed (US National Library of Medicine) and Google Scholar using the keywords “ticks” and “Taiwan,” supplemented with a perusal of references in these papers. We searched for potential theses with the keyword “ticks” (both in Chinese and English) in the National Digital Library of Theses and Dissertations in Taiwan. Books were also searched for in Google Scholar using the keywords “ticks” and “Taiwan” (both in Chinese and English), and by identifying references in related papers.

### Statistical analyses

When comparing frequency of tick infestation on migratory vs resident birds, a Chi-square test was applied in SPSS version 19.0 (Armonk, NY: IBM Corp.).

## Results

Between September 2014 and April 2016, the seven study sites were mist-netted for a total of 56 times, with each site surveyed 2–17 times (Chongde: 2; Donghwa: 12; Buluowan: 2; Xibao: 5; Lianhua Pond: 10; Luoshao: 17; Hehuan Farm: 8). A total of 4145 captures of 3096 individuals of 86 bird species (including 74 individuals of wounded or accidently trapped birds) were examined for tick infestations. Of these, 2455 individuals were captured only once, and the remaining 641 individuals were captured 2–8 times. These birds included 2406 individuals of 55 resident species and 690 individuals of 31 migratory species (Additional file 1: Table S1). Only larval (but not nymphal and mature) ticks were collected, and a total of 19 larval ticks were recovered from 4 of these 3096 bird individuals, with a mean load of 0.006 ticks/individual and a prevalence of 0.13%. These ticks belonged to *Haemaphysalis doenitzi* and *Ixodes columnae*, and were collected from one resident *Sinosuthora webbiana* (with 14 *H. doenitzi*), one resident *S. webbiana* (with one *I. columnae*), one migratory *Emberiza spodocephala* (with one *I. columnae*) and one migratory *Turdus pallidus* (with 3 *I. columnae*). The mean load of ticks was the same in resident birds (0.006 ticks/individual) as in migratory birds (0.006 ticks/individual). Prevalence of tick presence was more than three times higher in migratory birds (0.29%) than in resident birds (0.08%), although the difference was not statistically significant (Chi-square test: *χ* ^2^ = 2.0, *df* = 1, *P* = 0.16).

Overall, 139 ticks collected from birds, comprising 48 larvae, 35 nymphs, 55 adults and one individual of unknown life stage, were examined. These included 19 larval ticks collected between September 2014 and April 2016 in eastern Taiwan, and 120 ticks opportunistically collected from two avian studies implemented during 1995–2008 around Taiwan (83 ticks collected from 1268 bird individuals; mean load of 0.065 ticks/individual) and 2009–2013 in eastern Taiwan (37 ticks from 6343 bird individuals; mean load of 0.006 ticks/individual). A total of 11 species of four genera (*Amblyomma* spp*.*, *H. doenitzi*, *H. flava*, *H. formosensis*, *H. hystricis*, *H. ornithophila*, *H. wellingtoni*, *I. columnae*, *I. granulatus*, *I. nipponensis*, *I. turdus* and *Rhipicephalus haemaphysaloides*) were identified, including three newly recorded species in Taiwan (*H. wellingtoni*, *I. columnae* and *I. turdus*) and six species collected from birds in Taiwan for the first time (*H. flava*, *H. formosensis*, *H. hystricis*, *I. granulatus*, *I. nipponensis* and *R. haemaphysaloides*). The identity of the three newly recorded species has been validated with 100% identity to the nucleotide sequence deposited in GenBank (*H. wellingtoni*: AB819221; *I. columnae*: AB819233; *I. turdus*: AB819259). Ticks were collected from 19 bird species, including seven migratory species, notably the thrush family (*Turdus chrysolaus*, *Turdus hortulorum*, *Turdus pallidus* and *Zoothera dauma*) (Table 2). *Haemaphysalis doenitzi* was the most common species (42 ticks), comprising > 30% of all collected ticks, followed by *H. ornithophila* (21 ticks), *H. wellingtoni* (17 ticks) and *I. columnae* (17 ticks) (Table 2). These four species accounted for nearly 70% of all ticks. In comparison, *I. columnae* infested the most diverse host species (9 species), followed by *I. turdus* (4 species) and *H. doenitzi* (3 species) (Table 2). A few immature ticks (16 individuals) could at best be identified to genus (*Amblyomma*, *Ixodes* or *Haemaphysalis* species) based on morphology and molecular methods (Table 2).

**Table 2:** Species of hard ticks (Ixodidae) and their bird hosts studied from 1995 to 2016 in Taiwan

Tick species	No. of ticks	Avian host species (no. of birds the ticks were removed from; abundance of tick life stages)
Genus *Amblyomma*		
*Amblyomma* spp*.*	6	*Turnix suscitator* (1; 6L)
Genus *Haemaphysalis*		
*Haemaphysalis doenitzi*	42	*Centropus bengalensis* (10; 3N, 16A); *Sinosuthora webbiana* (1; 14L); *Phasianus colchicus* (2; 2L, 7A)
*Haemaphysalis flava* ^b^	1	*Turdus pallidus* ^c^ (1; 1N)
*Haemaphysalis formosensis* ^b^	2	*Turdus hortulorum* ^c^ (1; 1N); *Zoothera dauma* ^c^ (1; 1N)
*Haemaphysalis hystricis* ^b^	12	*Pomatorhinus musicus* (1; 7N); *Z. dauma* ^c^ (3; 5N)
*Haemaphysalis ornithophila*	21	*Lophura swinhoii* (1; 4N); *T. pallidus* ^c^ (1; 1A); *Z. dauma* ^c^ (7; 16A)
*Haemaphysalis wellingtoni* ^a^	17	*Centropus sinensis* (1; 3L, 3N, 11A)
*Haemaphysalis* spp*.*	6	*Alcippe morrisonia* (1; 1N); *C. bengalensis* (1; 1N); *Otus spilocephalus* (1; 1L); *Parus monticolus* (1; 1L); *P. colchicus* (1; 1N); *Z. dauma* ^c^ (1; 1L)
Genus *Ixodes*		
*Ixodes columnae* ^a^	17	*A. morrisonia* (1; 2L); *Horornis acanthizoides* (1; 1?); *Emberiza spodocephala* ^c^ (1; 1L); *Ficedula hyperythra* (1; 1L); *L. swinhoii* (1; 3L); *S. webbiana* (2; 2L); *Tarsiger indicus* (1; 2L); *T. pallidus* ^c^ (2; 4L); *Yuhina brunneiceps* (1; 1L)
*Ixodes granulatus* ^b^	5	*E. spodocephala* ^c^ (4; 1L, 3N); *T. pallidus* ^c^ (1; 1A)
*Ixodes nipponensis* ^b^	1	*Phylloscopus fuscatus* ^c^ (1; 1N)
*Ixodes turdus* ^a^	4	*Anthus hodgsoni* ^c^ (1; 1A); *Prinia inornata* (1; 1N); *Turdus chrysolaus* ^c^ (1; 1N); *T. pallidus* ^c^ (1; 1A)
*Ixodes* spp.	4	*Tarsiger johnstoniae* (1; 1L); *Locustella alishanensis* (1; 1N); *Cyanoderma ruficeps* (1; 1L); *T. pallidus* ^c^ (1; 1L)
Genus *Rhipicephalus*		
*Rhipicephalus haemaphysaloides* ^b^	1	*T. chrysolaus* ^c^ (1; 1A)

*Abbreviations*: *L* larva, *N* nymph, *A* adult

^a^Newly recorded species in Taiwan

^b^First record on birds in Taiwan

^c^Migratory species

We found 10 papers containing information on hard ticks of birds in Taiwan (Table 3). A total of five genera and 16 species of hard ticks were identified based on the current and past studies, including nine species not previously discovered in Taiwan. The genus *Haemaphysalis* (9 species) was most represented, followed by *Ixodes* (5 species); each of the *Dermacentor* and *Rhipicephalus* contained one species and *Amblyomma* ticks could not be identified to the species level (Table 3).

**Table 3:** Lists of species of hard ticks (Ixodidae) and their bird hosts known to occur in Taiwan

Tick species	Avian host species	Source
Genus *Amblyomma*		
*Amblyomma* spp.	*Turnix suscitator*	[51]; this study
Genus *Dermacentor*		
*Dermacentor taiwanensis*	*Bambusicola thoracica*	[47]
Genus *Haemaphysalis*		
*Haemaphysalis bispinosa*	*Gallus gallus*	[46]
*Haemaphysalis doenitzi*	*Bambusicola thoracica*; *Centropus bengalensis*; *Psilopogon nuchalis*; *Sinosuthora webbiana*; *Phasianus colchicus*; *Pomatorhinus musicus*; *Zoothera dauma* ^c^	[52]; this study
*Haemaphysalis flava* ^b^	*Turdus pallidus* ^c^	This study
*Haemaphysalis formosensis* ^b^	*Turdus hortulorum* ^c^; *Z. dauma* ^c^	This study
*Haemaphysalis hystricis* ^b^	*Pomatorhinus musicus*; *Z. dauma* ^c^	This study
*Haemaphysalis mageshimaensis*	*Hypsipetes amaurotis*; *Zosterops japonicus*	[44]
*Haemaphysalis ornithophila*	*Arborophila crudigularis*; *Lophura swinhoii*; *T. pallidus* ^c^; *Z. dauma* ^c^	[43, 45, 46]; this study
*Haemaphysalis wellingtoni* ^a^	*Centropus sinensis*	This study
*Haemaphysalis yeni*	*C. bengalensis*	[48]
*Haemaphysalis* spp.	*Alcippe morrisonia*; *C. bengalensis*; *Otus spilocephalus*; *Parus monticolus*; *P. colchicus*; *Turnix suscitator*; *Z. dauma* ^c^	This study
Genus *Ixodes*		
*Ixodes columnae* ^a^	*A. morrisonia*; *Horornis acanthizoides*; *Emberiza spodocephala* ^c^; *Ficedula hyperythra*; *L. swinhoii*; *S. webbiana*; *Tarsiger indicus*; *T. pallidus* ^c^; *Yuhina brunneiceps*	This study
*Ixodes granulatus* ^b^	*E. spodocephala* ^c^; *T. pallidus* ^c^	This study
*Ixodes kuntzi*	*Sitta europaea*	[41, 42, 46]
*Ixodes nipponensis* ^b^	*Phylloscopus fuscatus* ^c^	This study
*Ixodes turdus* ^a^	*Anthus hodgsoni* ^c^; *Prinia inornata*; *Turdus chrysolaus* ^c^; *T. pallidus* ^c^	This study
*Ixodes* spp.	*Tarsiger johnstoniae*; *Locustella alishanensis*; *Cyanoderma ruficeps*; *T. pallidus* ^c^	This study
Genus *Rhipicephalus*		
*Rhipicephalus haemaphysaloides* ^b^	*T. chrysolaus* ^c^	This study

^a^Newly recorded species in Taiwan

^b^First record on birds in Taiwan

^c^Migratory species

A total of 85 ticks were individually assayed for pathogen infection, including 5 *Amblyomma* spp*.*, 24 *H. doenitzi*, 1 *H. flava*, 1 *H. formosensis*, 9 *H. hystricis*, 6 *H. ornithophila*, 8 *H. wellingtoni*, 3 *Haemaphysalis* spp., 17 *I. columnae*, 4 *I. granulatus*, 1 *I. nipponensis*, 5 *I. turdus*, and 1 *R*. *haemaphysaloides*. One *Anaplasma* species (*Anaplasma* sp. clone BJ01), one *Babesia* species (*Ba. microti*), two *Borrelia* species (*Bo. valaisiana* and *Bo. turdi*), one *Ehrlichia* species (*Ehrlichia* sp. BL157‐9) and three *Rickettsia* species (*R. conorii*, *R. helvetica* and *R. monacensis*) were successfully sequenced from six tick species (Table 4). *Rickettsia helvetica*, or a closely related species, was most frequently identified (8 times, all from the tick *I. columnae*), followed by *Bo. valaisiana* (3 times from *I. granulatus*) and *Bo. turdi* (twice from *I. turdus*). The other five pathogen species were detected only once (Table 4). Two-thirds of the 18 detections of pathogens were on ticks collected from migratory birds, particularly the pale thrush (*T. pallidus*) (Table 4).

**Table 4:** Pathogen species or closely related species identified in hard ticks (Ixodidae) collected from birds from 1995 to 2016 in Taiwan

Pathogen species	Tick species (no. of detections in different life stages)	Avian host of ticks (no. of ticks)
Protozoa		
*Babesia microti*	*Ixodes granulatus* (1L)	*Emberiza spodocephala* ^c^ (1)
Bacteria		
Order Spirochaetales		
Family Spirochaetaceae		
*Borrelia valaisiana*	*Ixodes granulatus* (1L, 2N^a^)	*E. spodocephala* ^c^ (3)
*Borrelia turdi*	*Ixodes turdus* (1N, 1A)	*Turdus chrysolaus* ^c^ (1)*; Turdus pallidus* ^c^ (1)
Order Rickettsiales		
Family Anaplasmataceae		
*Anaplasma* sp. clone BJ01	*Haemaphysalis ornithophila* (1N)	*Lophura swinhoii* (1)
*Ehrlichia* sp. BL157-9	*Haemaphysalis flava* (1N)	*T. pallidus* ^c^ (1)
Family Rickettsiaceae		
*Rickettsia conorii*	*Haemaphysalis ornithophila* (1A)	*Zoothera dauma* ^c^ (1)
*Rickettsia helvetica*	*Ixodes columnae* (8L^b^)	*Alcippe morrisonia* (1)*; Ficedula hyperythra* (1)*; Lophura swinhoii* (1); *Tarsiger indicus* (1)*; T. pallidus* ^c^ (4)
*Rickettsia monacensis*	*Ixodes nipponensis* (1N)	*Phylloscopus fuscatus* (1)

*Abbreviations*: *L* larva, *N* nymph, *A* adult

^a^The three *Ixodes granulatus* were removed from three different *Emberiza spodocephala*

^b^Three of the eight *Ixodes columnae* were removed from the same individual *Turdus pallidus*

^c^Migratory species

## Discussion

This is one of the few studies focusing on bird-derived ticks and their pathogens in Southeast Asia, showing 11 tick species, of which *H. wellingtoni*, *I. columnae* and *I. turdus* are new records for Taiwan, and *H. flava*, *H. formosensis*, *H. hystricis*, *I. granulatus*, *I. nipponensis* and *R. haemaphysaloides* have been collected from birds in Taiwan for the first time (but had been previously collected from mammals). In addition, eight pathogens have been detected in these ticks, among which *Bo. turdi*, *Anaplasma* sp. clone BJ01, *Ehrlichia* sp. BL157-9, *R. helvetica* and *R. monacensis* have not previously been identified in Taiwan. Migratory birds were found to host infected ticks and may play a role in disseminating pathogens. Our study demonstrates the paucity of information on ticks of birds and emphasizes the need for more research on ticks of birds in Taiwan.

*Haemaphysalis wellingtoni*, *I. columnae* and *I. turdus* have not previously been recorded in Taiwan, and this could be due to the limited research on ticks of birds, rather than a rare occurrence of these tick species in Taiwan, because birds are the primary hosts of these three species [62] and these ticks were repeatedly collected from birds or infested birds in large numbers (Table 2). For example, 17 *H. wellingtoni* were found on a resident, ground-foraging coucal *Centropus sinensis* in a small islet (Kinmen) near mainland China. *Ixodes columnae* has been found on various bird species in Taiwan, including both resident and migratory birds. *Ixodes turdus* was collected from four avian species: three migratory birds and one resident bird. This tick species has also been found on migratory birds in Japan [63] and Korea [20, 27], suggesting that migratory birds can potentially disperse *I. turdus* across countries.

The ticks *H. flava*, *H. formosensis*, *H. hystricis*, *I. granulatus*, *I. nipponensis* and *R. haemaphysaloides* have previously been found feeding on mammals in Taiwan, but not on birds. Mammals are the predominant hosts of these six tick species, although *H. flava* and *I. granulatus* can also be collected from birds [62]. Indeed, except for *H. hystricis* and *I. granulatus*, the other four species were rarely collected from birds (less than two tick individuals) (Table 2). Less frequent occurrence on birds and the lack of research on ticks of birds in Taiwan help explain why these six tick species were not previously found on birds. *Haemaphysalis flava* was collected from several mammal species in Taiwan, including boars, deer and dogs [42], and this species has been repeatedly collected from birds in Japan, particularly the migratory true thrushes (*Turdus* spp.) and buntings (*Emberiza* spp.) [64]; likewise, we found a nymphal *H. flava* on a migratory *T. pallidus*. Both *H. formosensis* and *H. hystricis* have been collected from mammals in Taiwan, particularly rodents [50]. Our study reveals that migratory birds are also the hosts of *H. formosensis*; in addition, we collected 12 immature *H. hystricis* from birds, demonstrating that birds might not be occasional hosts of *H. hystricis*, as previously considered [62]. *Ixodes granulatus* is one of the most abundant and widespread tick species in Taiwan and infests a diverse set of rodent species [48, 50, 65]; in this study, five *I. granulatus* ticks have also been collected from migratory birds, suggesting that birds might also serve as major hosts of these generalist ticks. By contrast, there are very few records of *I. nipponensis* in Taiwan, with two adults recovered from cattle, and *I. nipponensis* was not definitely confirmed to be native to Taiwan [48]. Our study verifies that *I. nipponensis* did occur in Taiwan, although it was retrieved from a migratory bird and might not have yet been established in Taiwan. *Ixodes nipponensis* is distributed in temperate broadleaf and mixed forests [62] and is a common species in Japan [66] and Korea [67, 68]. Taiwan’s subtropical climate may not be ideal for the subsistence of *I. nipponensis*, hence the failure to establish itself in Taiwan. *Rhipicephalus haemaphysaloides* is also a common ectoparasite on rodents of Taiwan [65]. Similar to *H. hystricis*, birds are deemed to be occasional hosts of *R. haemaphysaloides* [62], and indeed, we found only one adult *R. haemaphysaloides* on a migratory, ground-feeding *T. chrysolaus*.

In this study, *Amblyomma* ticks collected from a resident bird species could not be identified to species based on both molecular and morphological characteristics. In Taiwan, four *Amblyomma* species (*A. cordiferum*, *A. geoemydae*, *A. helvolum* and *A. testudinarium*; [48]) have been identified, among which, birds are known to be hosts of *A. geoemydae* and *A. testudinarium* [62]. Whether the ticks collected in this study belong to *A. geoemydae* or *A. testudinarium* needs further investigation.

Based on published studies on ticks of birds in Taiwan, a total of five genera and 16 species of hard ticks were found feeding on birds according to the current and past studies (Table 3). Among these, only seven species have previously been documented, and nine species were first identified in Taiwan based on this single study (Table 3). It is thus expected that more species will be discovered after further research on avian ectoparasites. Based on morphological characteristics, *Amblyomma* spp., *Haemaphysalis campanulata* and *Haemaphysalis phasiana* were reported to be collected from birds [51]. We identified those specimens through morphology and DNA sequences but confirmed them to be *Amblyomma* spp., *H. ornithophila* and *H. doenitzi*, respectively. *Haemaphysalis campanulata* and *H. phasiana* were thus excluded from the final list. However, it is suspected that *H. phasiana* might be synonymous to *H. doenitzi* (see remarks by [62]). Indeed, 16S rDNA sequences retrieved from the GenBank database showed 99.5% (400/402) similarity between *H. doenitzi* (GenBank: JF979402) collected in China [69] and *H. phasiana* (AB819220) collected in Japan [70]. Likewise, 16S rDNA sequences of the eight *H. doenitzi* assayed in this study were 96.5–98.5% similar to the *H. phasiana* sequence archived in GenBank. Whether *H. phasiana* is synonymous with *H. doenitzi* should be resolved when more genetic data (e.g. 12S rDNA sequence on *H. phasiana* lacking in GenBank) on both species are available.

Only 19 ticks were collected during the 2014–2016 study compared with 120 ticks collected from 1995 to 2013. A further analysis reveales that the mean load of ticks in eastern Taiwan is the same during 2014–2016 as for the 2009–2013 period (both mean load = 0.006 ticks/individual), but the mean load is more than 10-fold higher when birds were captured around Taiwan (mean load of 0.065, during 1995–2008 period) than when birds were captured in eastern Taiwan. The reason for much lower mean tick loads in eastern Taiwan remains to be investigated. In addition, only larval ticks were found during the 2014–2016 study, whereas ticks collected during 1995–2013 were composed primarily of nymphs and adults (29 larvae, 35 nymphs, 55 adults). Because nymphal and adult ticks are larger than the larvae (which are more difficult to notice), the difference in composition of the life stages could be due to ticks being thoroughly searched for during 2014–2016 but only opportunistically collected during 1995–2013. Therefore, it should be emphasized that the 1995–2013 collection is biased toward nymphs and adults, and occurrence of larvae is underestimated.

Ticks are primary vectors for the five parasitic genera (*Anaplasma* [71]; *Babesia* [72]; *Borrelia* [73]; *Ehrlichia* [71]; *Rickettsia* [2]) identified in ticks in this study. Among the six tick species in which eight microbial species have been detected (Table 4), only *H. ornithophila* does not bite humans [62], so people in Taiwan are at risk of infection from most of the identified tick-borne pathogens. In addition, although *H. ornithophila* feeds primarily on birds [62], other generalist ticks might help bridge the pathogen transmission from birds to humans.

Among the eight pathogens detected in ticks, *Ba. microti*, *Bo. valaisiana* and *R. helvetica* have been detected in larval ticks (Table 4). Relative to *R. helvetica*, which can be vertically transmitted in ticks [74], transovarial transmission of *Ba. microti* and *Bo. valaisiana* has rarely been documented in ticks [75, 76]. This suggests that birds might be reservoirs of *Ba. microti* and *Bo. valaisiana*, and larval ticks can be infected when feeding on birds. Indeed, birds were shown to be the reservoirs of *Bo. valaisiana* [77, 78]. By contrast, although *Ba. microti* has been detected in ticks collected from birds (e.g. [26, 79]), birds are not considered to be the reservoirs of *Ba. microti* [80]. Our study nevertheless suggests that birds might play a role in the maintenance of *Ba. microti*, although the possibility that the larval tick acquires the protozoan via co-feeding ticks cannot be ruled out.

Five pathogens, i.e. *Bo. turdi*, *Anaplasma* sp. clone BJ01, *Ehrlichia* sp. BL157-9, *R. helvetica* and *R. monacensis*, have not previously been identified in Taiwan. *Borrelia turdi* was first characterized in *I. turdus* on migratory *E. spodocephala* in Japan [81, 82], and later in *I. turdus* and *I. nipponensis* on migratory birds in Korea [27]. This spirochete has also been detected in ticks collected primarily from *Turdus* spp. birds in Europe, including Belgium [83], Norway [84], Poland [85], Portugal [86] and Spain [76, 87]. Birds have been demonstrated to be the reservoirs of *Bo. turdi* [78]. In the current study, we showed that *Bo. turdi* also occurred in Taiwan, and similarly, the spirochete was detected in *I. turdus* collected from migratory birds (*T. chrysolaus* and *T. pallidus*), suggesting that *Bo. turdi* might be spread by migratory birds, particularly the thrush. Nevertheless, although *I. turdus* can infest humans [62], until now, *Bo. turdi* has not been found to cause Lyme borreliosis in humans [88].

*Anaplasma* and *Ehrlichia* are rickettsiae belonging to the family *Anaplasmataceae* and are the causative agents of several emerging human and animal diseases [89, 90]. *Anaplasma* sp. clone BJ01 was first isolated from *Haemaphysalis longicornis* in China (GenBank: JN715833). This bacterium is closely related to uncultured *Anaplasma* spp. in Korea and USA; nevertheless, a high degree of dissimilarity in 16S rRNA sequences with known *Anaplasma* species might warrant its reclassification under a new genus [91]. *Anaplasma phagocytophilum* and a novel *Anaplasma* sp. have been detected in bird tissues [92–94], indicating that birds could potentially infect ticks with *Anaplasma*. *Ehrlichia* sp. BL157-9 was first identified in *Hyalomma asiaticum* from China [95], and was closely related to *Ehrlichia* sp. ERm58 in the *Ehrlichia canis* group recognized in *Rhipicephalus muhsamae* from Mali [96]. Likewise, *Ehrlichia chaffeensis* and an *Ehrlichia* species closely related to *E. canis* have been detected in birds [92, 93], implying that birds might be reservoirs of *Ehrlichia*. Whether *Anaplasma* sp. clone BJ01 and *Ehrlichia* sp. BL157-9 are pathogenic to humans remains to be determined.

*Rickettsia helvetica* is a tick-borne SFG rickettsia first characterized in *Ixodes ricinus* from Switzerland and later identified in several European countries [2]. While less reported, evidence of human or tick infection by *R. helvetica* has also been found in Asia, including Japan [97–99], Thailand [100], Laos [101] and Sakhalin Island of Russia [102]. A strain similar to *R. helvetica* has also been isolated from raccoon and sika deer in Japan [103, 104]. In this study, a strain closely related to *R. helvetica* has been repeatedly detected in *I. columnae* recovered from both migratory and resident birds, demonstrating that the potentially pathogenic *R. helvetica* [105] might have become established in Taiwan. This should concern physicians in Taiwan, particularly when *I. columnae* also bite humans [62]. Moreover, the fact that *I. columnae* has only been collected from birds in Taiwan and birds are potential reservoirs of *R. helvetica* [106, 107] emphasizes the need for more research on birds, their associated ticks and their effects on public health.

*Rickettsia monacensis* also belongs to SFG and was first isolated from *I. ricinus* in Germany [108]. This species is widespread in Europe and can cause disease in humans [105]. Rickettsiae closely related to *R. monacensis* were later identified from ticks in East and Southeast Asia, including China [109, 110], Korea [111] and Thailand [112]. *Rickettsia monacensis* has also been detected in bird tissue [94]. Akin to the finding in Korea [111], this study isolated a strain genetically close to *R. monacensis* from *I. nipponensis*. Because *I. nipponensis* is distributed mainly in temperate regions [62], and in this study, *I. nipponensis* was retrieved from one migratory bird, *R. monacensis* was likely imported through bird migration. Recently, severe fever with thrombocytopenia syndrome (SFTS), an emerging infectious disease caused by the SFTS virus and with a high mortality rate, has been detected in *I. nipponensis* in Korea [113, 114]. The occurrence of *I. nipponensis* in Taiwan, despite being rare, should thus warrant further scrutiny.

The role of birds, particularly migratory birds, in the spread of ticks and tick-borne pathogens has received much more recognition in recent decades [7, 13, 115–117]. In this study, several ticks and tick-borne pathogens were discovered for the first time in Taiwan, including some that were found only on migratory birds (the tick *I. turdus*; the pathogens *Bo. turdi* and *Ehrlichia* sp. BL157-9), although it remains unclear if this is due to a lack of studies on ectoparasites of birds (namely, the same ticks and pathogens might be found in resident birds after thorough surveillance). In addition, while some ticks and pathogens were previously recognized in Taiwan, it is unknown whether the same species of ticks (e.g. *H. flava* and *H. formosensis*) and tick-borne pathogens (e.g. *Ba. microti* and *Bo. valaisiana*) recovered from migratory birds were acquired in Taiwan or from other countries (where ticks acquired pathogens and then were carried by migratory birds to Taiwan), so that a cross-country genetic mixture in ticks and pathogens is likely to occur. Therefore, the significance of migratory birds in the spread of exotic ticks and tick-borne pathogens in Taiwan, and whether these ticks and pathogens can become established in Taiwan, warrants further investigation. This is particularly true when the majority of migrant birds found in this study forage on the ground [118], which makes them more likely to acquire ticks compared with birds foraging in trees or shrubs [13, 29]. Studies are also needed on the seasonality of ticks in Taiwan to assess which life stage of which tick species is more active during the bird migration season (September to April of the following year) and thus more likely to be dispersed. In eastern Taiwan, it has been demonstrated that rodents are infested with more *R. haemaphysaloides* in October and November than in the other studied seasons, and larvae peak from October to January [49], but information on the seasonality of nearly all other tick species in Taiwan remains very limited.

## Conclusion

Our study demonstrates a paucity of knowledge on ticks of birds and their associated pathogens in Taiwan and Southeast Asia. Birds are capable of spreading ticks over long distances; moreover, pathogens harbored by ticks might differ when ticks were collected from birds vs mammals, with the latter much more frequently studied than the former in Taiwan. More research on ticks of birds is thus warranted, which can be facilitated with the integration of ornithologists in the studies of ticks.

## Additional file

Additional file 1: Table S1. Species of birds and number of captures and unique individuals mist-netted between September 2014 and April 2016 in eastern Taiwan. (DOCX 16 kb)
