# Supplementary material for: Tick-borne pathogens in ticks collected from birds in Taiwan
Source: Parasit Vectors. 2017 Nov 25;10:587. doi: 10.1186/s13071-017-2535-4 (PMC5702202; doi:10.1186/s13071-017-2535-4)
Supplement: Additional file 1: Table S1. — Species of birds and number of captures and unique individuals mist-netted between September 2014 and April 2016 in eastern Taiwan. (DOCX 16 kb) [file 13071_2017_2535_MOESM1_ESM.docx]

**S1** Species of birds and number of captures and unique individuals mist-netted between September 2014 and April 2016 in eastern Taiwan.

| Species | Individual | Capture | Number and species of ticks collected |
| --- | --- | --- | --- |
| *Abroscopus albogularis* | 26 | 26 | 0 |
| *Acridotheres javanicus* | 3 | 3 | 0 |
| *Acridotheres tristis* | 6 | 6 | 0 |
| *Acrocephalus orientalis** | 1 | 1 | 0 |
| *Aegithalos concinnus* | 151 | 183 | 0 |
| *Alcippe morrisonia* | 148 | 175 | 0 |
| *Anthus cervinus** | 1 | 1 | 0 |
| *Anthus hodgsoni** | 70 | 81 | 0 |
| *Anthus trivialis** | 1 | 1 | 0 |
| *Bambusicola sonorivox* | 7 | 8 | 0 |
| *Brachypteryx montana* | 3 | 3 | 0 |
| *Calliope calliope** | 60 | 91 | 0 |
| *Caprimulgus affinis* | 1 | 1 | 0 |
| *Carpodacus formosanus* | 34 | 39 | 0 |
| *Chalcophaps indica* | 3 | 3 | 0 |
| *Charadrius dubius** | 4 | 4 | 0 |
| *Cyanoderma ruficeps* | 359 | 620 | 0 |
| *Delichon dasypus* | 7 | 7 | 0 |
| *Dendrocitta formosae* | 1 | 1 | 0 |
| *Dicrurus aeneus* | 2 | 2 | 0 |
| *Elanus caeruleus* | 3 | 3 | 0 |
| *Emberiza chrysophrys** | 1 | 1 | 0 |
| *Emberiza elegans** | 1 | 1 | 0 |
| *Emberiza pusilla** | 24 | 25 | 0 |
| *Emberiza rutila** | 2 | 2 | 0 |
| *Emberiza spodocephala** | 260 | 357 | 1 *Ixodes columnae* |
| *Emberiza tristrami** | 2 | 2 | 0 |
| *Enicurus scouleri* | 1 | 1 | 0 |
| *Erpornis zantholeuca* | 4 | 4 | 0 |
| *Ficedula hyperythra* | 6 | 6 | 0 |
| *Fringilla montifringilla** | 3 | 3 | 0 |
| *Fulvetta formosana* | 26 | 54 | 0 |
| *Gallirallus striatus* | 1 | 1 | 0 |
| *Garrulus glandarius* | 1 | 1 | 0 |
| *Heterophasia auricularis* | 11 | 12 | 0 |
| *Hirundo rustica** | 3 | 3 | 0 |
| *Hirundo tahitica* | 8 | 9 | 0 |
| *Horornis acanthizoides* | 81 | 107 | 0 |
| *Horornis borealis** | 39 | 45 | 0 |
| *Horornis fortipes* | 9 | 9 | 0 |
| *Hypothymis azurea* | 15 | 15 | 0 |
| *Hypsipetes leucocephalus* | 3 | 3 | 0 |
| *Ixobrychus cinnamomeus* | 1 | 1 | 0 |
| *Jynx torquilla** | 1 | 1 | 0 |
| *Lanius bucephalus** | 1 | 2 | 0 |
| *Lanius schach* | 4 | 4 | 0 |
| *Liocichla steerii.* | 111 | 122 | 0 |
| *Locustella alishanensis* | 8 | 12 | 0 |
| *Locustella lanceolata** | 1 | 1 | 0 |
| *Locustella ochotensis** | 2 | 2 | 0 |
| *Lonchura atricapilla* | 6 | 6 | 0 |
| *Lonchura punctulata* | 22 | 22 | 0 |
| *Lonchura striata* | 122 | 148 | 0 |
| *Motacilla cinerea** | 2 | 2 | 0 |
| *Muscicapa ferruginea** | 8 | 8 | 0 |
| *Myophonus insularis* | 1 | 1 | 0 |
| *Nycticorax nycticorax* | 1 | 1 | 0 |
| *Phoenicurus auroreus** | 46 | 58 | 0 |
| *Phylloscopus borealis** | 24 | 32 | 0 |
| *Phylloscopus fuscatus** | 2 | 2 | 0 |
| *Pnoepyga formosana* | 1 | 3 | 0 |
| *Pomatorhinus musicus* | 25 | 44 | 0 |
| *Prinia flaviventris* | 39 | 84 | 0 |
| *Prinia inornata* | 9 | 16 | 0 |
| *Psilopogon nuchalis* | 5 | 5 | 0 |
| *Pycnonotus* spp. | 21 | 24 | 0 |
| *Pyrrhula erythaca* | 3 | 3 | 0 |
| *Rostratula benghalensis* | 2 | 2 | 0 |
| *Schoeniparus brunneus* | 2 | 2 | 0 |
| *Sinosuthora webbiana* | 285 | 573 | 14 *Haemaphysalis doenitzi*;1 *Ixodes columnae* |
| *Spizixos semitorques* | 39 | 43 | 0 |
| *Streptopelia chinensis* | 1 | 1 | 0 |
| *Streptopelia orientalis* | 1 | 1 | 0 |
| *Tarsiger johnstoniae* | 35 | 48 | 0 |
| *Trochalopteron morrisonianum* | 38 | 41 | 0 |
| *Troglodytes troglodytes* | 3 | 3 | 0 |
| *Turdus cardis** | 1 | 1 | 0 |
| *Turdus chrysolaus** | 25 | 27 | 0 |
| *Turdus eunomus** | 7 | 7 | 0 |
| *Turdus naumanni** | 4 | 4 | 0 |
| *Turdus obscurus** | 4 | 4 | 0 |
| *Turdus pallidus** | 89 | 100 | 3 *Ixodes columnae*^a^ |
| *Turnix suscitator* | 4 | 4 | 0 |
| *Yuhina brunneiceps* | 205 | 233 | 0 |
| *Zoothera dauma** | 1 | 1 | 0 |
| *Zosterops japonicus* | 492 | 526 | 0 |
| Total | 3096 | 4145 | 0 |

*migratory specie

^a^the three *Ixodes columnae* collected from the same *Turdus pallidus* individual all infected with *Rickettsia helvetica*
